# Case Report: Cavitary *Legionella pneumophila* pneumonia in a kidney transplant recipient: mNGS-guided diagnosis and prolonged combination therapy

**DOI:** 10.3389/fmed.2026.1697062

**Published:** 2026-03-12

**Authors:** Junhong Liu, Yu Zhou, Feilong Xu, Wei Liu, Huaizhou Chen, Qiang Yan, Junjun Guo, Liusheng Lai

**Affiliations:** Department of Organ Transplantation, 924th Hospital of the Chinese People’s Liberation Army Joint Logistic Support Force, Guilin, Guangxi, China

**Keywords:** antimicrobial therapy, atypical pneumonia, immunocompromised host, kidney transplantation, *Legionella pneumophila*, metagenomic next-generation sequencing

## Abstract

*Legionella pneumophila* is an uncommon but potentially life-threatening cause of pneumonia in solid organ transplant (SOT) recipients. Diagnosis is challenging due to nonspecific features and the limited sensitivity of conventional assays. Metagenomic next-generation sequencing (mNGS) offers unbiased detection and may be particularly valuable in immunocompromised hosts with refractory pneumonia. We report the first documented case in Asia of cavitary Legionella pneumonia in a kidney transplant recipient. A 60-year-old man presented with fever and bilateral pulmonary nodules 5 months post-transplant. Despite empirical antifungal and antibacterial therapy, his condition progressed radiologically to cavitary disease. Bronchoalveolar lavage fluid mNGS identified abundant *L. pneumophila* reads, confirming the diagnosis. Initial azithromycin monotherapy achieved transient improvement but failed to prevent radiological progression. Escalation to prolonged dual therapy with azithromycin and levofloxacin resulted in rapid symptomatic relief, progressive cavity regression on serial computed tomography, and preserved allograft function. Sequential blood-based mNGS demonstrated declining pathogen reads paralleling recovery. This brief research report emphasizes three practice points for SOT recipients with refractory pneumonia: (1) early mNGS can shorten time-to-diagnosis when routine tests are inconclusive; (2) Legionella infection may manifest with atypical cavitary lesions in immunocompromised hosts, warranting scheduled imaging even when symptoms improve; and (3) prolonged macrolide–fluoroquinolone combination therapy may be required for severe or non-resolving cases. Together with our literature review, this case expands understanding of the radiological spectrum, diagnostic strategies, and therapeutic considerations of Legionella pneumonia in transplant populations.

## Background

Solid organ transplant (SOT) recipients are highly susceptible to opportunistic respiratory infections due to prolonged immunosuppressive therapy, which compromises T-cell–mediated immunity and host defense against intracellular pathogens. Among atypical bacteria, *Legionella pneumophila* represents (2) an uncommon but clinically meaningful cause of pneumonia in this population. Although Legionella accounts for a small fraction of community-acquired pneumonia (3) in the general population, infection in SOT recipients is associated with disproportionately higher morbidity and mortality; reported case-fatality rates can reach 30–50% when diagnosis is delayed.

Early recognition in transplant patients remains challenging. Clinical manifestations such as fever, cough, and pulmonary infiltrates overlap substantially with bacterial and fungal pneumonias. Standard diagnostic assays have important limitations (13): urinary antigen testing predominantly detects (15) *L. pneumophila* serogroup 1, cultures require specialized media and are slow to yield results, and PCR assays are not universally available and may lack broad coverage. These constraints can prolong diagnostic delays, particularly in immunocompromised hosts, where timely initiation of targeted therapy is critical.

Radiological presentations in SOT recipients may diverge from classical patterns. While lobar consolidation and ground-glass opacities are typical in immunocompetent patients, immunosuppressed hosts may exhibit bilateral nodules, diffuse infiltrates, or cavitary lesions. Cavitary disease is especially rare and often raises concern for alternative etiologies such as tuberculosis or invasive fungal infection, further complicating the diagnostic process.

Metagenomic next-generation sequencing (mNGS) has emerged (4) as a powerful diagnostic modality, enabling unbiased pathogen detection directly from clinical specimens. In non-resolving or atypical pneumonia (12), mNGS can identify organisms missed by conventional tests and may provide semi-quantitative insights into pathogen burden. Despite its promise, published evidence on integrating mNGS findings into therapeutic decision-making and longitudinal disease monitoring in transplant recipients remains limited.

Here we report a kidney transplant recipient who developed cavitary pneumonia due to *L. pneumophila*, diagnosed through mNGS after conventional assays were inconclusive. This case underscores three clinically relevant issues: the diagnostic utility of early mNGS, the potential for atypical cavitary presentations in immunocompromised hosts, and the need for prolonged or combination antimicrobial therapy in severe disease while balancing graft-preserving immunosuppression. By situating the case within the broader literature, we aim to expand understanding of diagnostic and therapeutic considerations for Legionella pneumonia in transplant populations.

### Admission assessment (April 18, 2025; Day 0)

A 60-year-old man presented with a 1-week history of fever and sore throat. He had undergone allogeneic kidney transplantation 5 months earlier for chronic glomerulonephritis and was receiving oral tacrolimus 1.5 mg twice daily (bid), mizoribine (MZR) 50 mg bid, and methylprednisolone 8 mg once daily (qd). On admission, he had no dyspnea. Vital signs were: temperature, 36.0 °C; respiratory rate, 20 breaths/min; oxygen saturation, 99%. This decision was based on the initial radiologic pattern (bilateral nodular opacities) together with a positive serum (1→3)-β-D-glucan, while awaiting further microbiologic clarification. Given potential drug–drug interactions, tacrolimus exposure and renal function were closely monitored after dose adjustment, and early reassessment of clinical status and imaging was planned.

**Figure 1:**
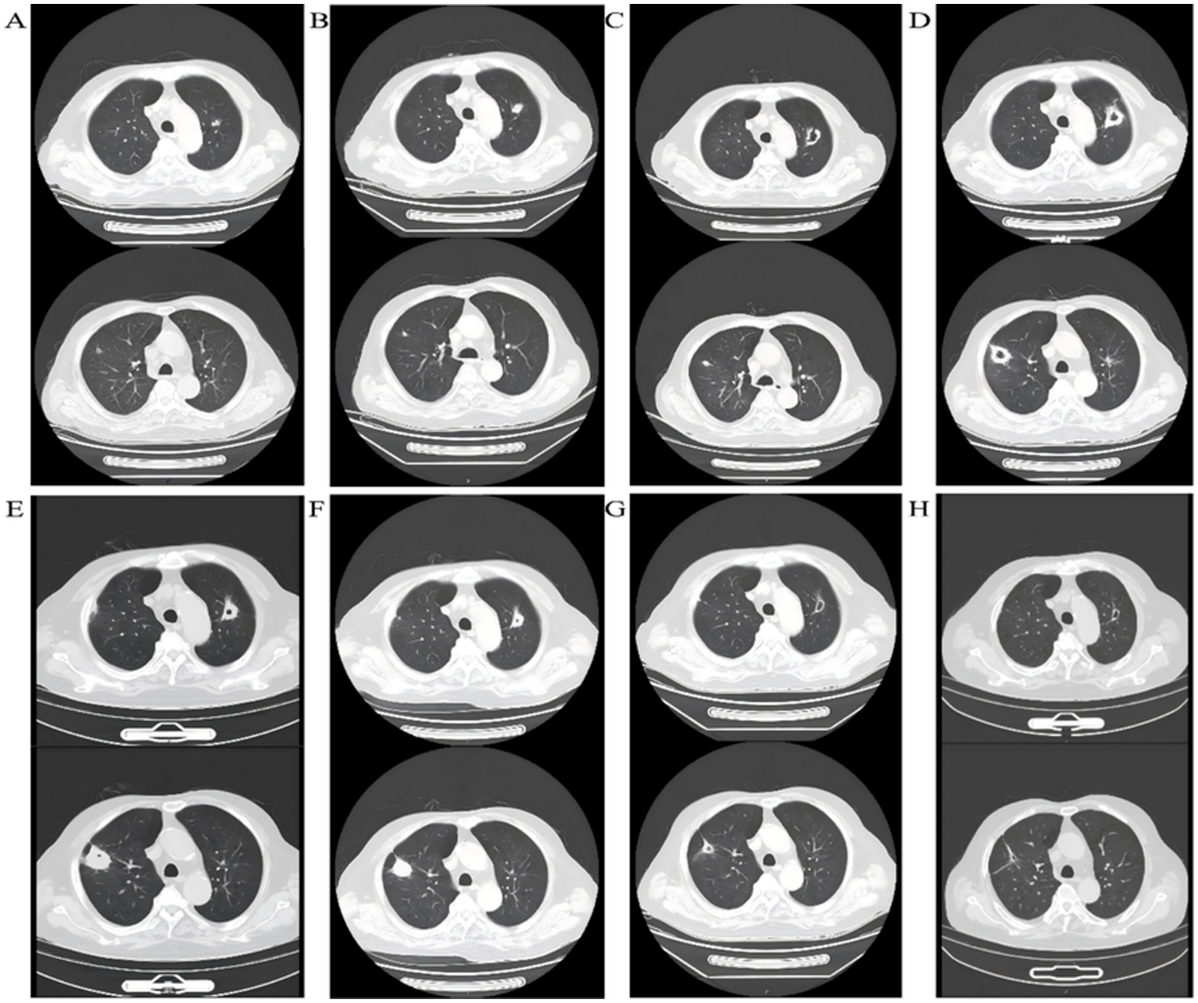
Sequential chest CT images of a kidney transplant recipient with *Legionella pneumophila* pneumonia. Representative scans **(A–H)** show disease progression from admission to follow-up: initial bilateral nodular infiltrates, development of ground-glass opacities and cavitation during azithromycin monotherapy, enlargement of cavities, and subsequent absorption and regression after prolonged azithromycin–levofloxacin combination therapy, with final imaging confirming resolution and preserved graft function.

Laboratory tests showed a white blood cell count of 3.01 × 10^9^/L, a CD3^+^ T-lymphocyte count of 554 cells/μL, C-reactive protein (CRP) of 28.8 mg/L, and serum creatinine (Cr) of 129 μmol/L. Serum (1→3)-β-D-glucan (BDG) was positive, and galactomannan (GM) was negative. Chest computed tomography (CT) revealed multiple bilateral nodular opacities (Figure 1A).

A pulmonary fungal infection was suspected. Empiric intravenous voriconazole 200 mg every 12 h (q12h) was initiated. Tacrolimus was reduced to 0.5 mg qd to limit immunosuppression. Early reassessment of imaging and microbiology was planned.

### Clinical progression and initial therapeutic adjustment (April 30, 2025; Day 12)

Fever persisted despite antifungal therapy (peak 38.5 °C). Repeat testing showed a white blood cell count of 3.32 × 10^9^/L, CRP of 129.48 mg/L, and a CD3^+^ T-lymphocyte count of 171 cells/μL. Chest CT revealed increased right lower-lobe ground-glass opacities (GGO) with radiological progression (Figure 1B), making a fungal etiology less likely.

**Figure 2:**
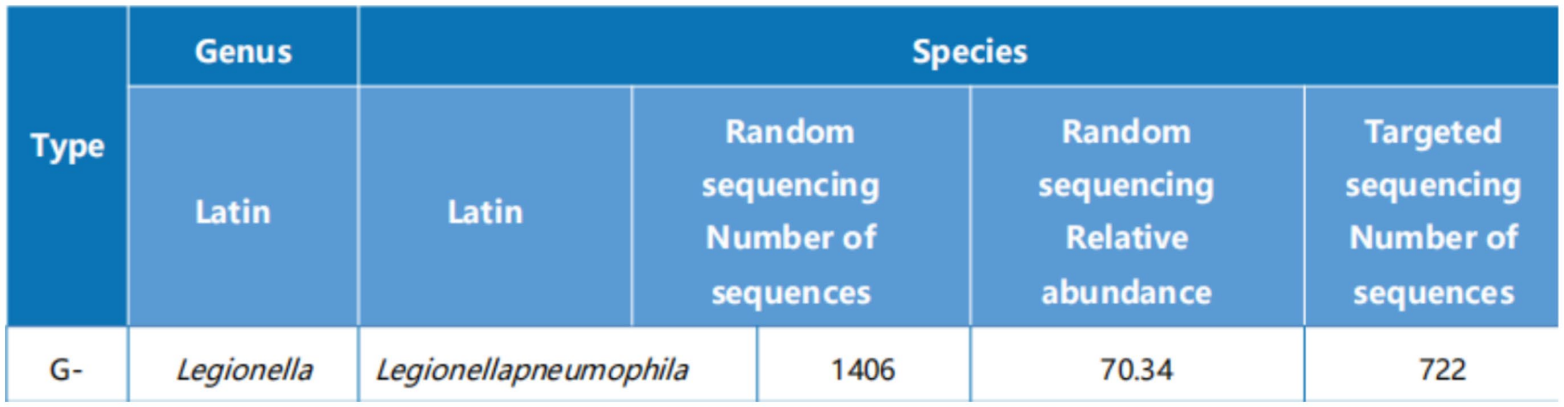
mNGS results of bronchoalveolar lavage fluid. *Legionella pneumophila* was identified with 1,406 reads in random sequencing (relative abundance 70.34%) and 722 reads in targeted sequencing, confirming it as the causative pathogen.

Empiric intravenous ceftriaxone 2 g qd was initiated, and MZR was discontinued. Bronchoscopy was performed, and bronchoalveolar lavage fluid (BALF) was submitted for mNGS. mNGS identified *L. pneumophila* with high read counts (722 sequence reads; Figure 2). Based on the clinical and radiological features, a diagnosis of *L. pneumophila* pneumonia was established. Therapy was switched to intravenous azithromycin 500 mg qd. Reassessment of symptoms, imaging, and inflammatory markers was scheduled within 1–2 weeks. The treatment switch was triggered by BALF mNGS identifying *Legionella pneumophila* with high abundance, which supported a targeted anti-Legionella regimen. In parallel, we planned scheduled reassessment of symptoms, inflammatory markers, and follow-up imaging, and we monitored immunosuppressant exposure and safety parameters during macrolide therapy.

### Targeted therapy and emergence of cavitation (May 13, 2025; Day 25)

After 10 days of targeted therapy, fever and systemic symptoms improved; however, chest CT demonstrated enlargement of several upper-lobe nodules with new cavitary lesions (Figure 1C).

**Figure 3:**
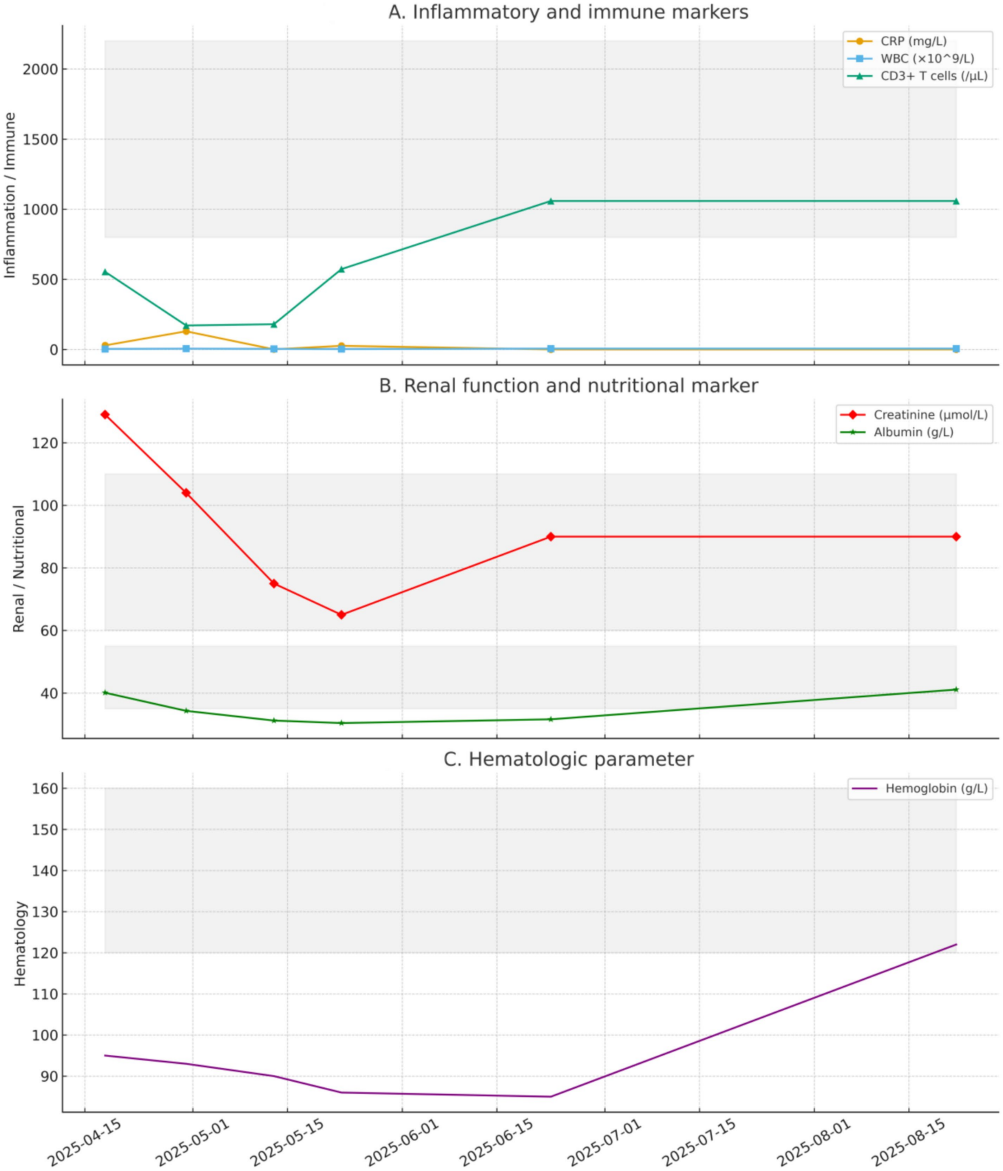
Temporal trends of laboratory parameters in a kidney transplant recipient with *Legionella pneumophila* pneumonia. **(A)** Inflammatory and immune markers (CRP, WBC, CD3^+^ T cells). **(B)** Renal function and nutritional marker (creatinine, albumin). **(C)** Hematologic parameter (hemoglobin). Gray shaded areas indicate reference ranges. Serial values are shown from admission (Day 0, 2025.04.18) to follow-up (Day 126, 2025.08.22).

Laboratory values were as follows: white blood cell count, 3.19 × 10^9^/L; CD3^+^ T-lymphocyte count, 181 cells/μL. Renal allograft function was stable (Cr, 75 μmol/L). Given symptomatic improvement and the short-term imaging lag typical of Legionella infection, the regimen was continued with close monitoring (see Figure 3).

### Prolongation and combination therapy (May 23, 2025; Day 35)

Around Day 21 of azithromycin therapy, the patient developed right-sided pleuritic chest pain with cough. Follow-up chest CT showed further enlargement of bilateral cavitary lesions (Figure 1D), suggesting an inadequate response to monotherapy.

Treatment was escalated to intravenous azithromycin 500 mg qd plus levofloxacin 400 mg qd. Escalation was justified by radiologic progression with enlarging cavitary lesions, suggesting an inadequate response rather than isolated imaging lag in an immunocompromised host. During combination therapy, we performed close safety monitoring, including renal allograft function, potential interactions affecting tacrolimus exposure, and class-related adverse effects (e.g., QT-related symptoms and other tolerability issues). No adverse events requiring discontinuation were recorded. One week later, symptoms improved further. On June 10, 2025 (Day 53), chest CT (Figure 1E) showed substantial resolution of lesions with reduction in cavity size; inflammatory markers declined, and clinical status improved. On June 23, 2025 (Day 66), after approximately 1 month of combination intravenous therapy, CT (Figure 1F) showed continued resolution. Renal allograft function remained stable, and the patient met discharge criteria.

### Follow-up

To consolidate the response and reduce relapse risk, oral azithromycin 500 mg qd plus levofloxacin 400 mg qd was continued. At 1 month (July 28, 2025), chest CT showed ongoing resolution with cavity regression (Figure 1G). Allograft function was stable, and clinical recovery persisted, so the regimen was maintained. At 2 months (August 22, 2025), chest CT (Figure 1H) showed further cavity regression without new symptoms, and the existing regimen was continued.

## Discussion

### Susceptibility and clinical characteristics

SOT recipients are highly susceptible (1) to opportunistic and atypical infections because of the sustained immunosuppression required to prevent allograft rejection. Long-term exposure to calcineurin inhibitors, antiproliferative agents, and corticosteroids leads to impaired T-cell—mediated immunity, reduced macrophage activity, and diminished cytokine signaling, compromising control of intracellular pathogens such as *L. pneumophila*. Although Legionella causes a minority of pneumonias in the general population, the burden in immunocompromised cohorts is disproportionately higher and associated with greater morbidity and mortality (6). The clinical presentation in transplant patients frequently overlaps (9) with bacterial or fungal pneumonia, including fever, cough, and dyspnea, while laboratory findings such as leukopenia or elevated CRP are nonspecific. This overlap explains why empirical therapy directed at Gram-negative bacteria or fungi may initially be chosen, as in our patient, and why therapeutic delays are common.

Atypical radiological manifestations further complicate recognition. While typical Legionella infection presents with lobar or multilobar consolidations and ground-glass opacities, transplant recipients may develop diffuse nodules, bilateral infiltrates, or cavitary lesions—findings rarely described in the general population. In our case, the emergence of multiple bilateral nodules initially raised suspicion for fungal infection, leading to empirical antifungal therapy. Only with progression to cavitation and identification of *L. pneumophila* by mNGS was the diagnosis established. This highlights the importance of maintaining a broad differential diagnosis in transplant patients and considering atypical pathogens when empirical therapy fails.

### Diagnostic challenges and the role of mNGS

Traditional diagnostic modalities for Legionella infection include urinary antigen testing (10), culture on buffered charcoal yeast extract agar, and PCR assays (5). While urinary antigen testing provides rapid results, it detects only *L. pneumophila* serogroup 1, missing infections caused by other serogroups or species. Culture is considered the reference standard yet is technically demanding, requires specialized media, and often yields false negatives due to prior antibiotic exposure or the organism’s fastidious nature. PCR offers enhanced sensitivity, but assay availability is inconsistent, and the range of detectable strains is limited. These limitations are particularly problematic in immunocompromised hosts, where early and precise pathogen identification is critical.

mNGS enables unbiased detection of a broad spectrum of pathogens directly from clinical specimens, circumventing the limitations of targeted assays. In our case, BALF mNGS revealed a high abundance of *L. pneumophila* sequences, allowing timely redirection of therapy from empirical antifungals to targeted antimicrobials. Beyond diagnostic accuracy, mNGS provides semi-quantitative insights into pathogen load: the high read counts in our patient suggested active infection, whereas subsequent reductions paralleled clinical and radiological improvement under combination therapy. Interpretation requires caution, however, as distinguishing colonization from infection and excluding contaminants remain challenges. Future integration of host-response profiling and microbial load kinetics may further refine clinical utility.

### Radiological cavitation: mechanisms and implications

Cavitary lung lesions are an unusual manifestation (14) of Legionella pneumonia. Cavity formation typically implies necrosis of lung parenchyma due to uncontrolled infection, vascular compromise, or secondary bacterial invasion. In immunocompromised hosts, several mechanisms may contribute: impaired immune clearance with persistent intracellular replication, a high bacterial burden overwhelming monotherapy, dampened granulomatous responses under long-term immunosuppression, and possible secondary colonization by other bacteria. In our patient, bilateral cavitary lesions developed after 3 weeks of azithromycin monotherapy despite symptomatic improvement, underscoring that clinical resolution does not necessarily equate to radiological recovery. Close imaging follow-up is essential, and recognition of cavitary disease should prompt consideration of therapeutic escalation and prolonged dual therapy.

### Therapeutic strategies in immunocompromised hosts

International guidance supports macrolides or fluoroquinolones as first-line (7) agents for Legionella pneumonia because both classes achieve high intracellular concentrations. In immunocompetent patients with mild disease, 7–10 days of monotherapy usually suffices. In immunocompromised hosts, particularly transplant recipients, longer courses—often 3–4 weeks—are required to achieve microbiological clearance and prevent relapse. The relative merits of macrolides versus fluoroquinolones are debated; macrolides offer immunomodulatory effects, whereas some observational data suggest fluoroquinolones may achieve faster defervescence (11). Given complementary properties, combination therapy is reasonable (8) for severe or refractory cases, despite the absence of large randomized trials. Our patient’s rapid improvement after adding levofloxacin to azithromycin supports this approach.

An additional consideration is drug–drug interactions with immunosuppressants. Macrolides inhibit cytochrome P450 3A4 and can raise tacrolimus levels; fluoroquinolones may also affect tacrolimus metabolism to a lesser extent. In our case, careful monitoring of tacrolimus trough concentrations allowed maintenance of stable graft function without adverse effects. Clinicians should remain vigilant and adjust dosing as needed.

### Prevention and follow-up

Preventive strategies include rigorous water-system monitoring and disinfection in healthcare facilities, as nosocomial outbreaks are frequently linked to contaminated supplies. At the patient level, avoiding exposure to high-risk sources such as poorly maintained humidifiers, hot tubs, or cooling towers is advisable. Radiological resolution is often delayed, particularly when cavitation occurs; serial CT at 4- to 6-week intervals can track healing and exclude complications such as persistent cavities, secondary infection, or bronchiectasis. In our patient, imaging at one and 2 months demonstrated progressive cavity regression, corroborating treatment efficacy. Ongoing surveillance of graft function is also essential.

### Broader implications of mNGS

Beyond Legionella, mNGS holds promise for diagnosing a wide spectrum of opportunistic infections in transplant recipients, including rare fungi, atypical bacteria, and viral reactivations often missed by conventional tests. Its ability to detect multiple pathogens simultaneously is particularly relevant in polymicrobial infections. Wider implementation will depend on addressing cost, turnaround time, and standardized reporting, but early incorporation into workups for refractory pneumonias in immunocompromised hosts may improve outcomes and antimicrobial stewardship.

### Strengths and limitations

Strengths of this report include dense longitudinal phenotyping with serial CT imaging and microbiologic support by BALF mNGS, enabling time-aligned therapeutic decisions and follow-up assessment. However, several limitations should be acknowledged. First, this is a single case and therefore not generalizable to all transplant recipients. Second, culture-based isolation and antimicrobial susceptibility testing were not available, limiting pathogen characterization beyond sequencing-based identification. Third, mNGS provides semi-quantitative outputs that may vary across laboratories and workflows; thus, results should be interpreted in clinical context rather than as a standalone endpoint. Future prospective cohorts are warranted to define optimal treatment strategies (including duration and the role of combination therapy) for severe or non-resolving Legionella pneumonia in solid organ transplant recipients.

## Data Availability

The original contributions presented in the study are included in the article/supplementary material, further inquiries can be directed to the corresponding authors.
